# Extracorporeal membrane oxygenation as a bridge to liver transplantation for acute respiratory distress syndrome-induced life-threatening hypoxaemia aggravated by hepatopulmonary syndrome

**DOI:** 10.1186/cc10476

**Published:** 2011-09-29

**Authors:** Antoine Monsel, Hervé Mal, Hélène Brisson, Rubin Luo, Daniel Eyraud, Corinne Vézinet, Chung Hi Do, Qin Lu, Jean-Christophe Vaillant, Laurent Hannoun, Pauline Houssel, François Durand, Jean-Jacques Rouby

**Affiliations:** 1 Multidisciplinary Intensive Care Unit, Department of Anesthesiology and Critical Care, La Pitié-Salpêtrière Hospital, Assistance Publique-Hôpitaux de Paris, UPMC Univ Paris 06, 83 Bd de l'Hôpital, 75013, Paris, France; 2 Department of Pneumology, Hopital Bichat-Claude Bernard, Assistance Publique Hôpitaux de Paris, 46 Rue Henri Huchard 75018, Paris, France; 3 Department of Hepatobiliary and Liver Graft Surgery, La Pitié-Salpêtrière Hospital, Assistance Publique-Hôpitaux de Paris, 83 Bd de l'Hôpital 75013, Paris, France; 4 Department of Hepatology, Hôpital Beaujon, Assistance Publique-Hôpitaux de Paris, 100 Bd Gen Leclerc 92110, Clichy, France; 5 Department of Emergency Medicine, Second Affiliated Hospital, Zhejiang University, School of Medicine, Hangzhou, 310009, China

**Keywords:** Acute respiratory distress syndrome, hepatopulmonary syndrome, hypoxaemia, extracorporeal membrane oxygenation, orthotopic liver transplantation

## Abstract

**Introduction:**

Combined with massive lung aeration loss resulting from acute respiratory distress syndrome, hepatopulmonary syndrome, a liver-induced vascular lung disorder characterized by diffuse or localized dilated pulmonary capillaries, may induce hypoxaemia and death in patients with end-stage liver disease.

**Methods:**

The case of such a patient presenting with both disorders and in whom an extracorporeal membrane oxygenation was used is described.

**Results:**

A 51-year-old man with a five-year history of alcoholic cirrhosis was admitted for acute respiratory failure, platypnoea and severe hypoxaemia requiring emergency tracheal intubation. Following mechanical ventilation, hypoxaemia remained refractory to positive end-expiratory pressure, 100% of inspired oxygen and inhaled nitric oxide. Two-dimensional contrast-enhanced (agitated saline) transthoracic echocardiography disclosed a massive right-to-left extracardiac shunt, without patent foramen ovale. Contrast computed tomography (CT) of the thorax using quantitative analysis and colour encoding system established the diagnosis of acute respiratory distress syndrome aggravated by hepatopulmonary syndrome. According to the severity of the respiratory condition, a veno-venous extracorporeal membrane oxygenation was implemented and the patient was listed for emergency liver transplantation. Orthotopic liver transplantation was performed at Day 13. At the end of the surgical procedure, the improvement in oxygenation allowed removal of extracorporeal membrane oxygenation (Day 5). The patient was discharged from hospital at Day 48. Three months after hospital discharge, the patient recovered a correct physical autonomy status without supplemental O_2_.

**Conclusions:**

In a cirrhotic patient, acute respiratory distress syndrome was aggravated by hepatopulmonary syndrome causing life-threatening hypoxaemia not controlled by standard supportive measures. The use of extracorporeal membrane oxygenation, by controlling gas exchange, allowed the performing of a successful liver transplantation and final recovery.

## Introduction

Extracorporeal membrane oxygenation (ECMO) has the capacity to support gas exchange as well as haemodynamics and is, therefore, a rescue therapeutic option for life-threatening respiratory and/or cardiac failure. ECMO has been tested in acute respiratory distress syndrome (ARDS) [1,2] and before and after lung transplantation [3]. Another potential use of ECMO could be the management of life-threatening hypoxaemia in patients with hepatopulmonary syndrome (HPS), selected for orthotopic liver transplantation (OLT). HPS, a liver-induced vascular lung disorder, is characterized by diffuse or localized dilated pulmonary capillaries and, less commonly, pleural and pulmonary arteriovenous communications coexisting with normal alveolar ventilation [4]. Hypoxaemia is usually responsive to oxygen therapy [4], but OLT appears as the only successful treatment [5,6]. However, in the most severe forms of HPS (PaO_2 _< 50 mmHg on room air), OLT is associated with increased respiratory morbidity/mortality due to frequent post-operative worsening of hypoxaemia [5,7]. One case of successful use of ECMO in the management of life-threatening hypoxaemia following OLT for HPS has been reported [8] but there are no data concerning its use in the pre-operative management.

## Materials and methods

We here report the use of ECMO as a bridge to OLT in a patient with refractory hypoxaemia resulting from combined ARDS and HPS.

## Results

### Diagnosis of hepatopulmonary syndrome

A 51-year-old man was admitted to our institution for haematemesis in a context of acute alcoholic intoxication. He had a five-year history of alcoholic cirrhosis with several episodes of bleeding from œsophageal varices and one episode of acute alcoholic hepatitis treated by corticosteroids. No hypoxaemia was detected during his medical follow-up and successive hospitalizations. On admission (Day 1), he was in respiratory failure with platypnoea. His arterial oxygen saturation (SaO_2_) before intubation was 87% at rest in a supine position while breathing 15 L/minute oxygen (O_2_) via a high concentration O_2 _face mask. Immediate tracheal intubation and mechanical ventilation (MV) support were required. On MV, pH, PaCO_2 _and PaO_2_, were 7.29, 77 mmHg, and 58 mmHg, respectively, using 100% O_2_, a tidal volume (TV) of 6 ml/kg of ideal body weight and a positive end-expiratory pressure (PEEP) of 10 cmH_2_O. Inhaled nitric oxide at 40 parts per million did not result in any improvement in gas exchange. Chest radiograph demonstrated moderate bilateral pleural effusion without clear evidence of alveolar consolidation and the diagnosis of HPS was suspected. Two-dimensional contrast-enhanced (agitated saline) transthoracic echocardiography disclosed a hyperkinetic systolic profile with low left ventricular filling pressures. The main abnormality was a massive right-to-left extra-cardiac shunt, without patent foramen ovale, confirming the diagnosis of HPS.

### Diagnosis of acute respiratory distress syndrome

**Figure 1:**
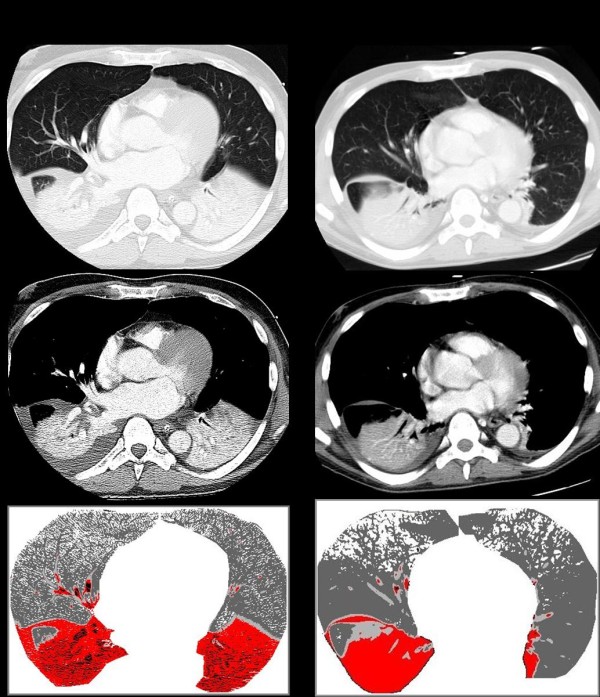
**Chest CT performed on Day 3 and Day 37 after the onset of refractory hypoxaemia**. **a) **Chest computed tomography (CT) on Day 3 following the onset of refractory hypoxaemia showing a bilateral consolidation of lower lobes with a right pleural effusion extending to the main fissura (upper CT section, parenchymal window). Large vessels are opacified within each lower lobe consolidation (middle CT section, mediastinal window). The application of a colour encoding system (lower part of the figure) confirms the consolidation of lower lobes [red colour, characterizing nonaerated lung regions with CT attenuations > -100 Hounsfield densities (HU)], shows the hypervascularization of lung consolidations (black colour, characterizing pulmonary vessels containing contrast material with CT attenuations > +200 HU) and evidences the hyperinflation of upper lobes (white colour, characterizing hyperinflated lung regions with CT attenuations < -900 HU). Normally (dark grey colour, characterizing lung regions with CT attenuations ranging between -900 and -500 HU) and poorly aerated lung regions (light grey colour, characterizing lung regions with CT attenuations ranging between -500 and -100 HU) are located to upper lobes. **b) **The same CT sections obtained at Day 20 following liver transplantation (37 days after the onset of refractory hypoxaemia) showing a persisting consolidation of the right lower lobe without pleural effusion. Large vessels are not anymore opacified within the consolidation (middle CT section, mediastinal window). The application of the colour encoding system (lower part of the figure) confirms the consolidation, the presence of hyperinflated lung regions in upper lobes. It does not show anymore hypervascularization within the right consolidation, suggesting a regression of HPS.

Contrasting with bedside anterior chest radiography, contrast computed tomography (CT) of the thorax demonstrated bilateral consolidations of lower lobes with dilated pulmonary vessels, moderate right pleural effusion and no evidence of pulmonary embolism (Figure 1a). The diagnosis of focal ARDS was made according to previously described criteria [9]. Secondary analysis of CT sections with the colour encoding system of the software Lungview^® ^(Institut National des Télécommunications, Evry, France) [10,11], confirmed the presence of enlarged pulmonary vessels within consolidations, suspected on mediastinal windows (Figure 1a). Quantitative analysis of the whole lung according to a technique previously described [12], demonstrated an increase in lung tissue (938 mL) predominating in the upper lobes (578 mL), a massive loss of lung aeration predominating in the lower lobes (99 mL of gas) and the presence of hyperinflation of the upper lobes (463 mL), a pattern strongly evocative of focal acute respiratory distress syndrome [13]. Beside lung failure, the first 24 hours after admission were marked by the development of circulatory shock requiring norepinephrine, acute renal failure and acute liver failure with cell necrosis: aspartate amino transferase (ASAT) = 1340 IU/L, alanine aminotransferase (ALAT) = 600 IU/L, alkaline phosphatase (AP) = 104 IU/L, gamma-glutamyl transpeptidase (γ-GT) = 80 UI/L, total bilirubin = 60 μmol/L, prothrombine time (PTT) = 40%, platelet count = 147 G/L. Despite the negativity of all bacteriological samplings, an empiric broad-spectrum antibiotic therapy was urgently started given the high suspicion of pneumonia.

### Extracorporeal membrane oxygenation

Over the next seven days, function of the involved organs stabilized with the exception of the lung, gas exchange remaining precarious (pH: 7.47, PaCO_2_: 40 mmHg, and PaO_2_: 51 mmHg under 100% FiO_2_). According to the severity of the respiratory condition, the French network for organ sharing national review board was petitioned and the patient was listed for transplantation with an urgent United Network for Organ Sharing (UNOS) 1a status. Facing the life-threatening hypoxaemia with failure of conventional ventilatory support to control gas exchange, even though he was given a high priority status, the patient was considered to be a candidate for ECMO as a bridge to transplantation. At Day 8, a veno-venous (V-V) ECMO was percutaneously placed with a 24-French venous drainage cannula in the right jugular vein, and a 19-French venous return cannula in the right femoral vein. According to previous recommendations demonstrating the superiority of polymethylpentene oxygenators over polypropylene oxygenators [14,15], the ECMO device consisted of the Medtronic Carmeda heparin-bound system, a Quadrox PLS 2050^® ^oxygenator (Maquet GmbH, Rastatt, Germany), and a Rotaflow RF32^® ^centrifugal pump (Maquet GmbH, Rastatt, Germany), a flow probe and 3/8-in. internal diameter heparin-bound tubing. Because of the heparin-bound tubing sets, systemic administration of heparin was limited to an intravenous bolus of 75 IU/kg before cannulation. Human albumin (5%, 500 mL) with physiological saline (500 mL) supplemented with 1,000 IU of heparin was used as a priming solution. Correct position of both cannulae was verified by transœsophageal echocardiography. Under the ECMO support, controlled MV using a constant inspiratory flow was used with a TV of 4 ml/kg, a respiratory frequency of 22/minute, a PEEP of 10 cmH_2_O and FIO_2 _60%. The objective was to maintain ECMO flow above 3 to 4 L/minute without ever reducing it below 1 L/minute to avoid the risk of cannula clotting.

### Liver transplantation

The patient remained stable under ECMO support until he underwent, after six days on a waiting list, uncomplicated OLT from a deceased donor on Day 13, (ECMO Day 5). During that period, the platelet count remained between 100 and 130 G/L. The procedure was performed under V-V ECMO. It required seven units of packed red blood cells, four units of fresh frozen plasma, eight units of human albumin (4%, 500 mL), and one liter of colloid. Cold and warm ischemic times were 400 minutes and 41 minutes, respectively. At the end of the surgical procedure, the improvement in oxygenation allowed removal of ECMO (Day 13). Despite an episode of methicillin resistant *Staphylococcus epidermidis *ventilator-associated pneumonia treated intravenously by glycopeptids, nitric oxide could be stopped on Day 20. On the same day, a CT of the chest demonstrated a marked improvement as compared with the pre-operative CT. As shown in Figure 1b, the left lower lobe was completely re-aerated whereas the right lower lobe appeared atelectatic, free of enlarged pulmonary vessels. Quantitative analysis of the whole lung revealed a decrease in lung tissue (from 938 to 815 mL), a partial re-aeration of the lower lobes (from 99 to 591 mL) and the persistence of some degree of hyperinflation in the upper lobes (from 463 to 213 mL). Transthoracic lung ultrasound revealed a limited hemi-diaphragm excursion, suggesting post-surgical diaphragmatic dysfunction with corresponding passive right lower lobe atelectasis. On Day 27, the patient's trachea was successfully extubated. Beside lung function improvement, liver function also improved from Day 18 with normalization of liver enzymes and coagulation factors. On Day 30, laboratory parameters showed ASAT = 33 IU/L, ALAT = 42 IU/L, AP = 72 IU/L, γ-GT = 115 IU/L, total bilirubin = 10 μmol/L, PTT = 90%, platelet count = 238 G/L. At discharge from the intensive care unit (Day 36), the patient remained O_2 _dependent (5 L/minute) with an arterial blood gas demonstrating pH, PaCO_2_, and PaO_2 _at 7, 42, 35 and 92 mmHg, respectively. The patient was discharged from hospital at Day 48. Three months after hospital discharge, the patient recovered a correct physical autonomy status without supplemental O_2_.

## Discussion

This report describes the case of a patient with deep hypoxaemia resulting from the combination of acute respiratory distress and hepatopulmonary syndromes and refractory to conventional mechanical ventilation support. The life-threatening respiratory condition was markedly improved by ECMO, allowing successful OLT. To our knowledge, this is the first case report describing the use of ECMO as a bridge to liver transplantation in a cirrhotic patient with life-threatening hypoxaemia resulting from combined ARDS and HPS.

### Diagnosis of hepatopulmonary syndrome

HPS is defined as a triad of liver disease-induced portal hypertension, increased alveolar-arterial gradient on room air, and intrapulmonary vascular dilatation. The latter is evidenced by contrast-enhanced echocardiography detecting the presence of microbubbles injected into an upper extremity vein within the left atrium [4-6]. The overperfusion of normally aerated lung regions reduces ventilation/perfusion ratios and causes hypoxaemia. It can be moderate (PaO_2 _between 60 and 80 mmHg breathing room air) and well tolerated, or severe (PaO_2 _≤ 60 mmHg breathing room air), with progressive development of shortness of breath [6]. Platypnoea, defined by an increased dyspnoea when moving from supine to upright position, and orthodoexia, defined by a decrease in PaO_2 _> 5% or > 4 mmHg when moving the patient from supine to upright position, are characteristic features of HPS: both are related to upright position-induced blood flow redistribution to lung zones with prominent vascular dilatations, a phenomenon resulting from the absence of pulmonary vascular tone [16]. In our patient, hypoxaemia was not diagnosed during the medical follow-up preceding OLT, likely because hypoxaemia was moderate and well tolerated. At hospital admission, however, platypnoea was present, raising the possibility of HPS, a hypothesis confirmed by a positive transthoracic contrast-enhanced echocardiography.

### Causes of the patient's refractory hypoxaemia

The patient's life-threatening and refractory hypoxaemia was the result of three additive mechanisms: HPS, ARDS and MV-induced lung hyperinflation. The patient's ARDS was characterized by a massive loss of lung aeration, affecting exclusively the lower lobes, and an excess of inflammation predominating in the upper lobes. Such a lung morphology, found in a majority of patients meeting criteria for ARDS, is generally associated with arterial hypoxaemia poorly responsive to PEEP [11]. In our patient, HPS-induced vascular dilatation predominantly found in nonaerated lower lobes (Figure 1a), contributed to increase the pulmonary shunt and rendered hypoxaemia totally refractory to PEEP and inhaled nitric oxide.

In addition, focal lung morphology exposes patients to MV-induced lung hyperinflation [11,17]. Despite the reduction of TV to 6 ml/kg, lung hyperinflation is observed in one-third of patients with acute respiratory distress syndrome [18]. As observed in our patient, it predominates initially in non-dependent and caudal lung regions [19] and can lead to late air cysts and bronchiectasis in the same lung regions [20]. The immediate effect of MV-induced hyperinflation is to redistribute pulmonary regional blood flow away from upper lobes towards non-aerated lower lobes, thereby further increasing pulmonary shunt and worsening hypoxaemia. The presence of HPS-induced vascular dilatation in lower lobes is another aggravating factor explaining why patient's life-threatening hypoxaemia could be reversed only by ECMO.

### Treatment of hepatopulmonary syndrome

The medical options in the case of severe HPS-related hypoxaemia are limited. Patients with severe hypoxaemia at rest should receive continuous long-term low-flow oxygen therapy [6]. The benefit provided by Trendelenbourg positioning, insertion of a transjugular intrahepatic portosystemic shunt, embolisation or pharmacological treatments, including inhaled nitric oxide and nitric oxide inhibitors, have been reported but never confirmed in larger studies [6]. Several studies have demonstrated that HPS-induced severe hypoxaemia can reverse after OLT [7,21,22]. According to the 2004 European Respiratory Society recommendations, the indication of OLT is firm if PaO_2 _is between 50 and 60 mmHg but is discussed on an individual basis if PaO_2 _is below 50 mmHg [6]. In our patient, hypervascularization within lower lobes was not anymore visible on lung CT performed 20 days after OLT, suggesting partial regression of HPS. Three months after OLT, the patient was definitively weaned from nasal O_2_, suggesting total reversal of HPS in a delay similar to what has been previously reported [23].

### Indications of extracorporeal membrane oxygenation

ECMO may be deployed as arterio-venous ECMO providing both cardiac and respiratory support or as V-V ECMO, which only provides oxygenation [2]. A recent clinical report describes the case of a 12 year-old child who, in the early post-operative period following OLT, developed life-threatening hypoxaemia attributed to an exacerbation of sepsis-induced ARDS by the existence of a pre-transplantation HPS [8]. Facing the failure of conventional MV combined with inhaled nitric oxide to provide adequate control of oxygenation, the patient was considered to be a candidate for V-V ECMO. He remained 18 days on ECMO support, was successfully weaned and remained well for longer than one year post-transplantation. This report led us to consider that ECMO could be a potential therapeutic option in our patient and proposed as a bridge to liver transplantation.

It is unlikely that HPS alone, without concomitant loss of lung aeration may justify ECMO. In fact, the combination of massive loss of lung aeration and HPS-induced vascular dilatation in lower lobes with MV-induced hyperinflation in upper lobes led to life-threatening hypoxaemia refractory to any conventional treatment. In our patient, ECMO could, via a better control of gas exchange, lower the risk of hypoxaemia -related organ failure during waiting time before OLT and during the surgical procedure. Furthermore, ECMO initiated preoperatively could efficiently prevent HPS-related worsening of postoperative hypoxaemia.

Beside its potential benefits, ECMO may have some harmful effects. The use of an extracorporeal circuit, requiring systemic anticoagulation, and often leading to thrombocytopenia carries a potential risk of bleeding complications, especially in a cirrhotic patient who may already have thrombocytopenia and compromised liver function tests. Moreover, a higher incidence of infectious complications related to the intravascular insertion of the ECMO cannulae can be dreaded in a cirrhotic patient. More data concerning the safety issues associated with the use of ECMO in patients with HPS are, therefore, needed.

## Conclusions

In conclusion, we report the case of a patient with ARDS, aggravated by HPS and MV-induced hyperinflation, responsible for life-threatening hypoxaemia not controlled by standard supportive measures. The use of ECMO, by controlling gas exchange, allowed the performing of successful OLT. These preliminary data, therefore, suggest that ECMO could have a place in patients eligible for OLT. It provides the basis to test this strategy in a larger number of patients presenting a combination of acute parenchymal disease and HPS.

## Key messages

• Our report suggests that ECMO could have a place as a bridge to liver transplantation in patients presenting a combination of acute parenchymal disease and hepatopulmonary syndrome

• It provides the basis to test this strategy in a larger number of patients

## Abbreviations

γ-GT: gamma-glutamyl transpeptidase; ALAT: alanine aminotransferase; AP: alkaline phosphatise; ARDS: acute respiratory distress syndrome; ASAT: aspartate amino transferase; CT: computed tomography; ECMO: extracorporeal membrane oxygenation; HPS: hepatopulmonary syndrome; MV: mechanical ventilation; O_2_: oxygen; OLT: orthotopic liver transplantation; PEEP: positive end-expiratory pressure; PTT: prothrombine time; SaO_2_: arterial oxygen saturation; (V-V) ECMO: veno-venous ECMO.

## Competing interests

The authors of this manuscript have no conflicts of interest to disclose as described by Critical Care.

## Authors' contributions

HB, DE, CV, CHD, JCV, LH, PH and FD helped to draft the manuscript. AM, HM and JJR helped to draft the manuscript and were involved in the revision of the final version. RL helped to draft the manuscript and performed the secondary analysis of CT sections with the colour encoding system of the software Lungview^®^. All authors have read and approved the manuscript for publication.
